# The Effects of Quality of Reinforcement on the Resurgence of Responding

**DOI:** 10.3390/bs15111531

**Published:** 2025-11-10

**Authors:** Patrick W. Romani

**Affiliations:** Department of Pediatrics, University of Colorado School of Medicine, Aurora, CO 80045, USA; patrick.w.romani@ucdenver.edu; Tel.: +1-(720)-777-2996

**Keywords:** response resurgence, behavioral persistence, reinforcer potency, progressive ratio assessment, quality of reinforcement

## Abstract

The current study evaluated the effects of quality of reinforcement on the resurgence of target behavior responding. We defined higher- or lower-quality stimuli in terms of reinforcer potency, and specifically by the identified extent to which a reinforcer maintains responding at progressively greater response requirements. We first conducted a stimulus potency analysis to empirically derive higher- or lower-quality reinforcers using a progressive ratio schedule of reinforcement. We then conducted a human operant study within a three-condition resurgence evaluation with three children diagnosed with developmental disabilities. During Condition 1, target behavior responding led to higher- or lower-quality reinforcement according to a variable interval (VI) 30-s schedule of reinforcement. Following stable responding, Condition 2 delivered higher- or lower-quality reinforcement to the alternative behavior according to a VI 30-s schedule of reinforcement as target behavior responding was placed on extinction. During Condition 3, responding to both the target and alternative responses was placed on extinction. All three participants showed greater resurgence of the target behavior to a greater extent within the condition associated with higher-quality reinforcer delivery. We will discuss these results considering the importance of quality of reinforcement to both experimental and applied behavior analysis.

## 1. Introduction

Resurgence of target responding is defined as the recurrence of a previously extinguished response following changing reinforcement conditions (Lattal & Wacker, 2015; Lattal & St. Peter-Pipkin, 2009). Researchers commonly study resurgence using a three-condition analysis. During Condition 1, an organism accesses reinforcement for engaging in a target response in lieu of a second, alternative response. During Condition 2, reinforcers for the target response are withheld and now allocated to the alternative response. Finally, during Condition 3, reinforcement for both responses is withheld. The re-emergence of the previously extinguished target response during Condition 3 is evidence of resurgence. Although often a temporary phenomenon, the study of resurgence has increased because of its potential impact on clinical treatment programs targeting the problem behavior of neurodiverse youth using differential reinforcement procedures. For example, any increase in problem behavior occurrence could lead to caregiver or client injury. Indeed, applied research has documented the occurrence of resurgence following as many as 76% of applications of reinforcement-schedule thinning (Briggs et al., 2018; see also Muething et al., 2021; Volkert et al., 2009). Thus, continued investigation into the conditions under which resurgence occurs is critical for both experimental and applied behavior analysis.

Dimensions of reinforcement, such as rate of reinforcement and magnitude of reinforcement, contribute to the occurrence of resurgence (Fisher et al., 2022). For example, Craig et al. (2017) manipulated magnitude of reinforcement to evaluate its effects on resurgence. Rats first pressed a lever to access one pellet during Condition 1 of the resurgence paradigm described above. During Condition 2, the rats either received five pellets (higher magnitude) or one pellet (lower magnitude) contingent on lever pressing. Lever pressing under both conditions resulted in extinction during Condition 3. Results showed greater suppression of lever pressing within the component associated with the higher magnitude of reinforcement delivery during Condition 2. A higher rate of resurgence of lever pressing occurred during Condition 3 within the component associated with the higher magnitude reinforcer delivery. These findings have important translational implications as clinical programming often incorporates differential reinforcement programs that deliver a higher rate or higher magnitude of reinforcement following alternative behavior occurrence (Browning et al., 2022; Shahan et al., 2024). Indeed, similar treatment arrangements have been shown to contribute to greater resurgence of destructive behavior following successful treatment implementation (Fisher et al., 2018; Greer et al., 2024). There may be other dimensions of reinforcement that similarly influence resurgence and have great implications for clinical treatment of destructive behavior.

Another dimension of reinforcement commonly implicated within successful differential reinforcement programs is quality of reinforcement (Graff & Karsten, 2012; Mace & Roberts, 1993; Virues-Ortega et al., 2014). Applied research has evaluated the quality of reinforcement by studying choice or time allocation to different stimuli (Fisher et al., 1992; DeLeon & Iwata, 1996; Pace et al., 1985). Studies including non-human animals have replicated the effects of rate and magnitude of reinforcement on resurgence with quality of reinforcement (Shahan et al., 2024). Recently, Shahan and colleagues (2024) evaluated the effect of qualitatively distinct reinforcers on resurgence of responding among rats. Within a two-experiment study, they delivered a 5% sucrose solution (during Experiment 1) or a 45 mg food pellet (during Experiment 2) as the higher- or lower-quality reinforcers, respectively. The authors quantified quality by using the bias parameter of the matching law. Results showed that the rats participating in Experiment 1 demonstrated higher rates of resurgence than the rats participating in Experiment 2. The researchers concluded that quality, as a dimension of reinforcement, affects resurgence similarly to magnitude and rate.

Although resurgence of non-human animal responding appears to be influenced by quality of reinforcement, a study specifically isolating this variable within a human sample is absent from the extant literature. As previous evaluations of quality of reinforcement on resurgence have focused on non-human animals, a natural extension of this literature would be to evaluate resurgence following empirically derived higher- or lower-quality stimuli with humans in a laboratory setting. The purpose of the current investigation was to evaluate the resurgence of button pressing within a human operant experiment under extinction conditions when a relatively higher- or lower-quality reinforcer was previously delivered contingent on responding. We conducted this experiment to extend Shahan et al. (2024) to determine if the delivery of higher- or lower-quality stimuli, as measured within a progressive ratio schedule of reinforcement, resulted in differential resurgence of target responding within a human operants arrangement. 

## 2. Method

### 2.1. Participants

A recruitment flier was available in the clinic waiting area in which participants received therapeutic services. The flier provided a general description of the study, the goal of recruiting neurodiverse children, and the contact information for the First Author. The three participants in this study were the first three that responded to this recruitment flier.

Diego was a 14-year-old boy diagnosed with Autism Spectrum Disorder (Level 1 for both social communication and restricted and repetitive behaviors) and an unspecified depressive disorder. He spoke in complete sentences. The research team recruited Diego from a psychiatric outpatient clinic. Diego’s mother provided consent for him to participate in the project, and Diego provided assent to participate in the project.

Daniel was an 8-year-old boy diagnosed with Autism Spectrum Disorder (Level 1 for both social communication and restricted and repetitive behaviors) and disruptive mood dysregulation disorder. He spoke in complete sentences. The research team recruited Daniel from a psychiatric partial hospitalization program. Daniel’s mother consented to Daniel’s participation, and Daniel assented to participation in the project.

Tilly was a 9-year-old girl diagnosed with 22q11.2 deletion syndrome and a mild intellectual disability. She communicated using short sentences. The research team recruited Tilly from a psychiatric outpatient clinic. Tilly’s father provided consent for her to participate in the project, and Tilly provided assent to participate.

### 2.2. Setting and Materials

Experimental procedures were conducted in a clinic therapy room at a university-based hospital. Experimental sessions occurred for 1 h weekly for 3 months. Each clinic therapy room contained a table and four chairs on one side of the room, and preferred activities were placed on a padded mat on the other side of the room. We used an iPad application (Countee; Peic & Hernandez, 2015) that permitted frequency measurement of button pushing as the data collection instrument. The research team programmed colored buttons onto the Countee screen that served as the dependent variables for the current study.

### 2.3. Dependent Variables and Interobserver Agreement

The dependent variable for both phases in this experiment was pushing a button that said, “Button 1” on the left-hand side of the screen or a button that said, “Button 2” on the right-hand side of the screen. We intended for Buttons 1 and 2 to serve as analogues for problem behavior and an appropriate alternative behavior, respectively. We programmed the buttons to be different colors, as well. We asked participants to accurately identify each color prior to using it within the experiment. Resurgence was defined as an increase in target behavior responding from the last session in Condition 2 to the first session of Condition 3 (King et al., 2025). The Countee program recorded button pressing when participants put their finger on either the button representing the target response (Button 1) or the button representing the alternative response (Button 2). When a button press led to reinforcement, the researcher paused the session and allowed the participant to engage with the relevant stimulus. After the reinforcement period ended, the researcher started the session again so the participant could continue pressing either button. Sessions ended after 5 min with reinforcement time excluded. At the end of the pre-programmed 5 min session, the Countee produced a summary file that expressed the rate of button presses as target responses and alternative responses per minute. A second observer reviewed 33% of the summary files and compared data on button pressing to the data entered on the graph. An agreement was defined as the second observer concluding that the same number of target and alternative button presses occurred during the session. A disagreement was when the second observer concluded that a different number of target and alternative button presses occurred during the session. The agreement was 100% for all three participants. Using the same Countee summary files, procedural fidelity was calculated on researcher delivery of reinforcement. For 33% of summary files, a correct researcher response for accurate reinforcement delivery was when the summary file showed the researcher paused the session (indicating delivery of reinforcement) for the first response after one of the predetermined intervals was set to end. An incorrect researcher response was when the researcher did not pause the session for the first response after one of the predetermined intervals was set to end. Procedural fidelity averaged 97% (range, 95–100%) for Diego and was 100% for Daniel and Tilly.

### 2.4. Design and Analysis

This experiment was conducted within two phases. During Phase 1, we conducted a stimulus potency analysis within a progressive ratio schedule of reinforcement. During Phase 2, we conducted a resurgence evaluation within a multielement design using a multiple schedule arrangement to evaluate differences in response resurgence following higher- or lower-quality reinforcer delivery contingent on button pushing.

### 2.5. Procedures

**Stimulus Potency Analysis (Phase 1).** During the paired stimulus preference assessment (Fisher et al., 1992), the research team evaluated Diego’s preference for YouTube videos, board games, puzzles, vocal discussion, coloring, and LEGOs. Daniel’s preference assessment evaluated YouTube videos, coloring, LEGOs, magnetiles, a train set, and puzzles, and Tilly’s preference assessment evaluated YouTube videos, bristle blocks, coloring materials, puzzles, and toy dinosaurs. The research team identified these items via an interview with the participants and their caregivers. The paired stimulus preference assessment began with the researcher introducing each stimulus to the participant by modeling how to interact with the stimulus. Afterwards, the researcher presented two items to the participants with the instruction to “Pick one.” Once the participant selected one of the items, the researcher permitted 20 s access to it before restricting the item. The researcher did not interact with the participant during this 20 s period. At this point, another two items were presented to the participant following the same procedures. Every item was paired with every other item to evaluate relative stimulus preference for each participant. A total of fifteen choices occurred during the preference assessment. The results of Digeo’s preference assessment showed YouTube videos as most preferred (selected on 100% of trials) and LEGOs as least preferred (selected on 20% of trials). The results of Daniel’s preference assessment showed YouTube videos as most preferred (selected on 100% of trials) and puzzles as least preferred (never selected). Tilly’s preference assessment showed coloring as the most preferred stimulus (selected on 100% of trials) and dinosaurs as least preferred (never selected).

We evaluated the potency of each stimulus within a single operant arrangement. The progressive ratio assessment was conducted first with the higher-preferred stimulus and then with the lower-preferred stimulus for Diego and the lower-preferred stimulus then the higher-preferred stimulus for Daniel and Tilly. This procedural difference was a mistake by the researcher. Although results for each participant were uniform, this, nonetheless, introduces concern for order effects that should be investigated further in future research. At the beginning of each session, the researcher directed the participant to a desk with an iPad with one button displayed on the screen. The relevant stimulus was placed behind the iPad and the participant was told to press the button to earn the relevant item. Participants always transitioned to the desk willingly, and no behavioral problems occurred throughout the experiment. Prior to the session beginning, the researcher said, “When I press start, you can push the button as many or as few times as you want.” The researcher provided no other instructions and did not engage with the participant during the reinforcement interval. The session began when the researcher pressed “Start” on the iPad screen. The researcher delivered the relevant stimulus to the participant for 20 s after meeting the predetermined reinforcement schedule for that session. After this 20 s play period, the researcher said, “It’s my turn now” and removed the stimulus from the participant’s reach and said, “You can push the button as many or as few times as you want.” The ratio schedules of reinforcement were FR 1, FR 5, FR 10, FR 20, FR 50, FR 100, and FR 500, similar to Tustin (1994). When a period of two consecutive minutes passed without a button press, the session ended, and following two consecutive sessions in which this criterion was met, the assessment was terminated.

**Resurgence Analysis (Phase 2).** The second phase was the resurgence analysis phase. The analysis conducted in Phase 2 was done separately for the higher- and lower-quality reinforcers. For the higher-quality reinforcer evaluation, a red button and a green button served as the discriminative stimuli, and for the lower-quality reinforcer evaluation, an orange and purple button served as the discriminative stimuli. The red and orange buttons represented an analogue for the “target behavior,” which, in an applied setting, could be problem behavior and the green and purple buttons represented an analogue for the “alternative behavior,” which, in an applied setting, could represent an appropriate alternative response, like communication. The resurgence analysis consisted of three conditions. Throughout all three conditions, the participant was vocally directed to a table and targeted higher- or lower-quality reinforcers were placed immediately behind a tablet with the relevant colored buttons programmed in front of them. The research said, “You can push either button as many or as few times as you want,” and pressed start on the tablet. The researcher provided no other instructions and did not engage with the participant during the reinforcement interval. During the first condition, which was used to establish a history of reinforcement for the target response, a press to the target response led to access to the reinforcer for 20 s according to a VI 30 s schedule of reinforcement (Mace et al., 1997, Experiment 3). Presses to the alternative response resulted in extinction. This condition ended when visual analysis confirmed differentiated rates of button presses for at least five consecutive sessions and the rate of reinforcement was similar for those sessions. The second condition intended to establish a reinforcement history for the response that was placed on extinction during the first condition. In this condition, a press to the alternative response resulted in access to the reinforcer for 20 s according to a VI-30 s schedule of reinforcement. Presses to the target response resulted in extinction. In this way, we established a similar reinforcement history for both responses, thus controlling rate of reinforcement and making the quality of reinforcement the independent variable. After the same level of stability in rate of responding and reinforcement occurred for at least five consecutive sessions, we conducted the third condition in which presses to both buttons were placed on extinction to evaluate the resurgence of responding. This third condition ended when responding to both buttons occurred at or near zero. The discontinue criterion was selected based on previous human operant research studying resurgence (e.g., King et al., 2025).

## 3. Results

**Figure 1:**
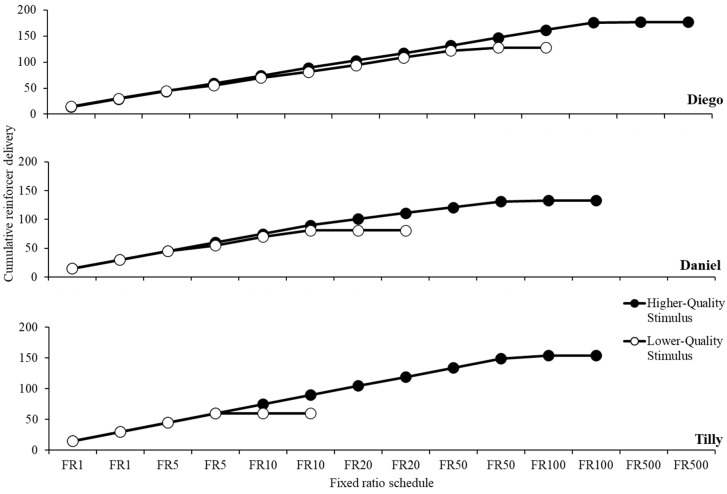
Stimulus potency analysis for Diego (top panel), Daniel (middle panel), and Tilly (bottom panel).

Figure 1 displays data from the stimulus potency analysis for the higher- and lower-preferred stimuli for Diego (top panel), Daniel (middle panel), and Tilly (bottom panel). The higher-preferred stimulus maintained button pressing until the FR-500 schedule of reinforcement, when button pressing decreased to zero or near-zero rates. In contrast, the lower-preferred stimulus maintained responding only until the FR-50 arrangement. Daniel’s stimulus potency analysis showed the higher-preferred stimulus maintained responding until the FR-100 arrangement. The lower-preferred stimulus maintained responding until the FR-20 arrangement. Finally, Tilly’s stimulus potency analysis showed the higher-quality stimulus maintained button pressing until the FR-100 arrangement. Responding when we delivered the lower-quality stimulus maintained until the FR-10 arrangement.

**Figure 2:**
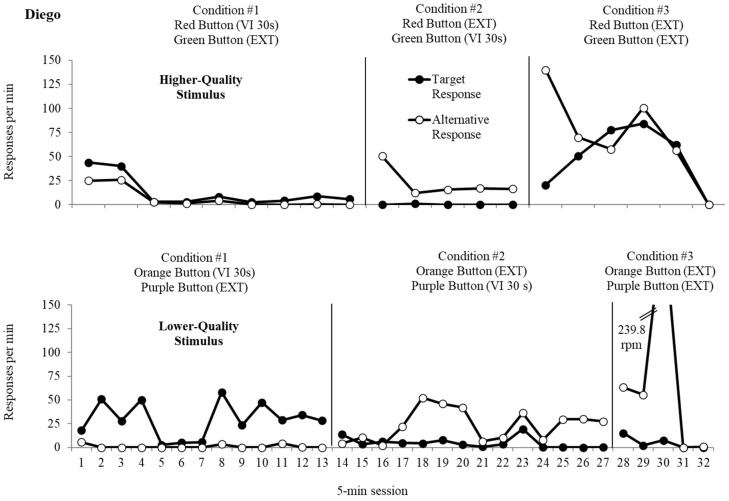
Resurgence analysis for Diego.

**Figure 3:**
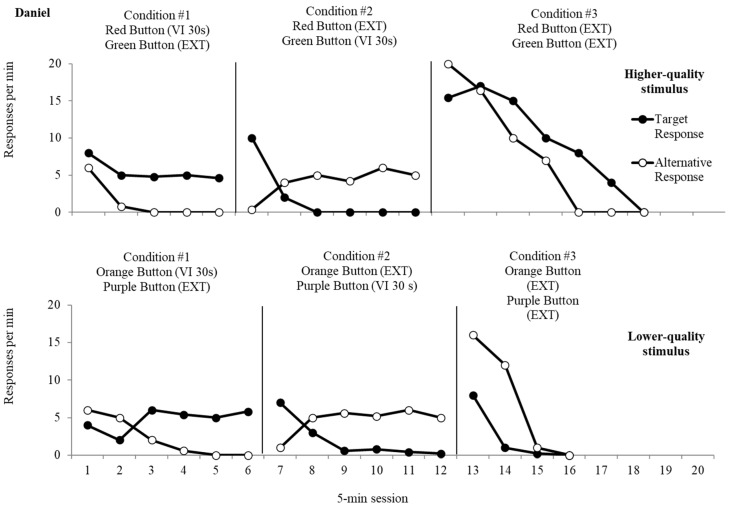
Resurgence analysis for Daniel.

**Figure 4:**
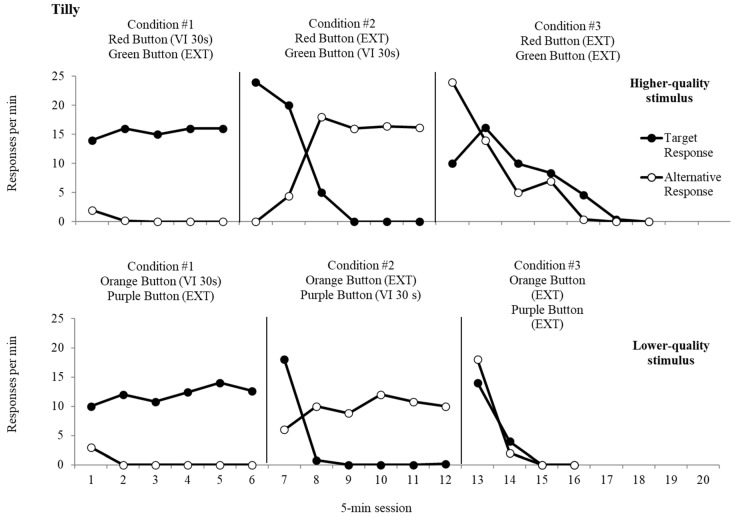
Resurgence analysis for Tilly.

The top panel of Figure 2, Figure 3 and Figure 4 displays data from the resurgence analysis for the higher-quality reinforcer for Diego, Daniel, and Tilly, respectively. Responses to the target response, which was initially programmed for reinforcement, produced button presses at an average rate of 14.3 responses per minute (rpm) for Diego, at an average rate of 5.5 rpm for Daniel, and at an average rate of 15.4 rpm for Tilly. Button presses to the alternative response, which was placed on extinction, resulted in an average rate of 7.6 rpm (Diego), 1.36 rpm (Daniel), and 0.44 rpm (Tilly). The average rate of reinforcement was 1.0 for the target responses, which was consistently obtained during Condition 1 for all three participants. During the second condition, responses to the alternative response led to an average rate of responding of 22.4 rpm (Diego), 4.1 rpm (Daniel), and 11.8 rpm (Tilly), and an average of 0.24 rpm (Diego), 2.0 rpm (Daniel), and 8.2 rpm (Tilly) for the target response. The average rate of reinforcement for responses to the alternative response was 1.0 reinforcers per minute, the same as Condition 1 for all three participants. Resurgence of target response presses occurred immediately during the third condition when both responses were placed on extinction (Diego *M* = 42.2 rpm; Daniel *M* = 9.9 rpm; Tilly *M* = 7.1 rpm). Responding decreased to zero after five sessions for Diego and after seven sessions for Daniel and Tilly. Alternative responses occurred at an average rate of 60.7 rpm (Diego), 7.6 rpm (Daniel), and 7.2 rpm (Tilly).

The bottom panel of Figure 2, Figure 3 and Figure 4 depicts data from the lower-quality stimulus’ resurgence analysis for Diego, Daniel, and Tilly, respectively. During the first condition, button presses to the target response resulted in an average of 29.3 rpm (Diego), 4.7 rpm (Daniel), and 12.0 rpm (Tilly), while responses to the alternative response, which resulted in extinction, remained low throughout the condition (Diego *M* = 1.1 rpm; Daniel *M* = 2.3 rpm; Tilly *M* = 0.5 rpm). The average rate of reinforcement was 1.0 for responses to the target response, the same as obtained during the first resurgence evaluation for all three participants. During the second condition, responses to the alternative response occurred at an average of 23.4 rpm (Diego), 4.6 rpm (Daniel), and 9.5 rpm (Tilly), and responses to the target response occurred at an average of 4.9 rpm (Diego), 2.0 rpm (Daniel), and 3.8 rpm (Tilly). The average rate of reinforcement for the alternative response was 0.9 reinforcers per min for Diego and 1.0 for Daniel and Tilly. During extinction, resurgence occurred at an average of 5.0 rpm (Diego), 2.3 rpm (Daniel), and 4.5 rpm (Tilly) for the target response. Responding decreased to zero after three sessions for Daniel and Tilly and four sessions for Diego for the target response. The alternative response persisted at an average rate of 71.7 rpm (Diego), 7.3 rpm (Daniel), and 5.0 rpm (Tilly).

We converted the extinction data into proportion of baseline for all three participants. Resurgence for target responses reinforced with the higher-quality reinforcer resulted in proportionally higher rates of responding when compared to the lower-quality reinforcer. When target behavior responding produced the higher-quality reinforcer, the average proportion of baseline during extinction was 3.16 for Diego, 1.81 for Daniel, and 0.46 rpm for Tilly. In contrast, when responding produced the lower-quality reinforcer, the average proportion of baseline was 0.17 for Diego, 0.49 for Daniel, and 0.37 rpm for Tilly. Data regarding persistence of alternative behavior responding was mixed for both participants. For Diego, average proportion of baseline for alternative behavior was 2.7 when the higher-quality reinforcer was delivered and 3.1 when the lower-quality reinforcer was delivered. Daniel demonstrated an average proportion of baseline of 1.9 for the higher-quality reinforcer and 1.5 for the lower-quality reinforcer, and Tilly demonstrated an average proportion of baseline of 0.61 for the higher-quality reinforcer and 0.52 for the lower-quality reinforcer.

## 4. Discussion

To date, human studies of resurgence have focused almost exclusively on the effects of rate and magnitude of reinforcement (Mace et al., 2010; Montague et al., 2025; Parry-Cruwys et al., 2011; Wacker et al., 2011). Comparatively few studies have evaluated the effects of any other dimension of reinforcement (e.g., quality of reinforcement) on changes in responding during extinction (Berg et al., 2015; Mace et al., 1997; Ringdahl et al., 2018). The primary purpose of the current study was therefore to evaluate how the quality of reinforcement affects the recurrence of button pressing. Results from Diego, Daniel, and Tilly revealed a higher level of resurgence of the target behavior following reinforcement with the higher-quality reinforcer, replicating Shahan et al. (2024) within a human operant arrangement. Taken together, these findings strengthen the case that reinforcement quality—like rate and magnitude (Craig et al., 2017; McComas et al., 2008; Montague et al., 2025; Shahan & Avellaneda, 2025)—plays a meaningful role in affecting resurgence.

One major challenge in studying reinforcement quality lies in how to objectively define and measure it. Stimuli identified as higher quality under low response requirements may not function as reinforcers under more effortful conditions (DeLeon et al., 1997; Tustin, 1994). Thus, evaluating reinforcer potency—that is, the conditions under which stimuli reliably maintain responding under more effortful conditions—provides one way to quantify relative quality (Roane, 2008). Potency may in fact be a more precise measure of quality than preference alone (Francisco et al., 2008; Glover et al., 2008; Penrod et al., 2008; Roane et al., 2001). Identifying participant preferences remains a useful first step, but preference may not be sufficient to determine quality. It seems reasonable to hypothesize that stimuli that maintain responding under high response requirements (i.e., higher-quality reinforcers) would have greater impact on strengthening the maintenance of treatment effects (Nevin & Wacker, 2013) than those that maintain responding under less-effortful conditions (i.e., lower-quality reinforcers). Overall, this effect was consistently shown in our study.

To date, there is a small but growing number of demonstrations of the impact that quality, as both a response and reinforcer dimension, can have on the persistence and resurgence of behavior. Although the results have been positive, these demonstrations have been translational studies and have often been conducted within human operant contexts. In an important exception, Weinsztok and DeLeon (2022) delivered higher- or lower-quality reinforcers contingent on an alternative and target behavior responding for autistic children receiving behavior-analytic services. In their study, the researchers compared a “Higher-Quality” condition that delivered a higher-quality reinforcer contingent on alternative behavior and a lower-quality reinforcer contingent on the target behavior to an “Equal Quality” condition in which the low-quality reinforcer was delivered following both alternative and target behavior responding. They systematically reduced treatment integrity, thereby introducing extinction for the alternative response. Interestingly, in contrast to the findings in Shahan et al. (2024) and the present experiment, their results showed that higher-quality reinforcers were associated with less resurgence of the target behavior. This could be due to the concurrent schedules arrangement in place, however. Either way, such discrepancies highlight the need for further investigation into how reinforcement quality translates across contexts and populations, particularly in applied settings.

Several limitations of the present study warrant consideration. First, while quality clearly influenced resurgence, the relative contribution of reinforcer potency remains unresolved. In this study, all three participants’ highest-preferred item was also shown to be the most potent reinforcer. This overlap is not always observed (e.g., Roane et al., 2001), suggesting the need for continued work disentangling preference from potency as distinct measures of quality. A logical follow-up study, following Nevin (1995), would be to conduct a parametric analysis of reinforcer potency and resurgence. For example, reinforcers that maintain responding at different levels of work could be systematically evaluated to determine when resurgence occurs and if these results vary across clinical subgroups. A similar limitation is the lack of a consistent demonstration of each participant’s higher- and lower-quality reinforcer throughout the experiment. This may be important as the relative quality of a stimulus can change over time. A third limitation of this study was the limited sample size (*n* = 3). An area for future research would be recruiting additional participants to establish the consistency of this finding within a human population. These data may support the translation of this work to applied settings. Relatedly, all three participants had diagnoses of neurodevelopmental disabilities—Diego and Daniel had similar presentations of Autism Spectrum Disorder and Tilly had mild intellectual disability and delayed language skills. It would be helpful for future research to replicate this study with participants with and without neurodevelopmental disabilities to evaluate the consistency of these findings.

In summary, this study recruited three participants diagnosed with developmental disabilities to examine whether higher- or lower-quality reinforcers differentially affected resurgence of a previously reinforced response. Results replicated previous research showing that quality of reinforcement is an important determinant of resurgence. These findings could have important implications for clinical settings that often rely on assessments of preference to obtain socially meaningful treatment results.

## Data Availability

The data presented in this study are available on request from the corresponding to author. The data are not publicly available due to privacy and ethical restrictions.
